# Marginal zone lymphoma-derived interfollicular diffuse large B-cell lymphoma harboring 20q12 chromosomal deletion and missense mutation of *BIRC3* gene: a case report

**DOI:** 10.1186/s13000-016-0588-x

**Published:** 2016-12-19

**Authors:** Joseph Hatem, April M. Schrank-Hacker, Christopher D. Watt, Jennifer J. D. Morrissette, Adam I. Rubin, Ellen J. Kim, Sunita D. Nasta, Mariusz A. Wasik, Agata M. Bogusz

**Affiliations:** 1 Department of Pathology and Laboratory Medicine, Hospital of the University of Pennsylvania, 7 E Gates Pavilion, 3400 Spruce Street, Philadelphia, 19104-4283 PA USA; 2 Department of Dermatology, University of Pennsylvania, Philadelphia, 19104 PA USA; 3 Department of Medicine, University of Pennsylvania, Philadelphia, 19104 PA USA

**Keywords:** Diffuse large B-cell lymphoma (DLBCL) with deletion 20q(del(20q)), Interfollicular diffuse large B cell lymphoma (DLBCL-IF), Marginal zone lymphoma

## Abstract

**Background:**

Diffuse large B-cell lymphoma (DLBCL) typically leads to effacement of the nodal architecture by an infiltrate of malignant cells. Rarely (<1%), DLBCL can present with an interfollicular pattern (DLBCL-IF) preserving the lymphoid follicles. It has been postulated that DLBCL-IF is derived from marginal zone B cells and may represent a large-cell transformation of marginal zone lymphoma (MZL), however no direct evidence has been provided to date. Here we describe a rare case of a diagnostically challenging DLBCL-IF involving a lymph node in a patient with a prior history of lymphadenopathy for several years and MZL involving skin.

**Case presentation:**

A 53-year old man presented to our Dermatology Clinic due to a 1-year history of generalized itching, fatigue of 2–3 month’s duration, nausea and mid back rash that was biopsied. PET (positron emission tomography)/CT (computed tomography) was performed and revealed inguinal, pelvic, retroperitoneal, axillary, and cervical lymphadenopathy. The patient was referred to surgery for excisional biopsy of a right inguinal lymph node.

Diagnostic H&E stained slides and ancillary studies were reviewed for the lymph node and skin specimens. B-cell clonality by PCR and sequencing studies were performed on both specimens.

We demonstrate that this patient’s MZL and DLBCL-IF are clonally related, strongly suggesting that transformation of MZL to DLBCL had occurred. Furthermore, we identified a novel deletion of the long arm of chromosome 20 (del(20q12)) and a missense mutation in *BIRC3* (Baculoviral IAP repeat-containing protein 3) in this patient’s DLBCL that are absent from his MZL, suggesting that these genetic alterations contributed to the large cell transformation.

**Conclusions:**

To our knowledge, this is the first report providing molecular evidence for a previously suspected link between MZL and DLBCL-IF. In addition, we describe for the first time del(20q12) and a missense mutation in *BIRC3* in DLBCL. Our findings also raise awareness of DLBCL-IF and discuss the diagnostic pitfalls of this rare entity.

## Background

Diffuse large B-cell lymphoma (DLBCL), the most common type of non-Hodgkin lymphoma, is defined as a neoplasm of large B lymphocytes that display a diffuse growth pattern [1]. DLBCL is a quite diverse group of malignancies with respect to cell morphology, pathogenesis, clinical presentation, and therapeutic response [1–3]. Gene expression profiling studies identified two major subgroups of nodal DLBCL based on the “cell of origin”: the germinal center (GC) B-cell-like DLBCL (GCB DLBCL) and the prognostically less favorable activated B-cell-like DLBCL (ABC DLBCL) [4]. Immunohistochemical signatures were developed to translate the molecular signatures and distinguish between the GCB DLBCL and non-GCB DLBCL [5].

The World Health Organization (WHO) classification distinguishes numerous subtypes of DLBCL [1]. In the majority of the cases, DLBCL leads to effacement of the nodal architecture by a diffuse infiltrate of malignant cells. Rarely, however, DLCBL can show an interfollicular pattern (DLBCL-IF) of proliferation, preserving the lymphoid follicles [6]. These cases constitute only about 1% of all DLBCL, frequently display a polymorphous appearance microscopically due to the admixture of non-neoplastic inflammatory cells, and often present a diagnostic challenge. Previous reports indicate that DLBCL-IF are predominantly of non-GCB type [6, 7]. Interestingly, the interfollicular large B cells in normal lymph node also show a non-GCB phenotype and share some immunophenotypic characteristics with monocytoid B cells [8]. It has been postulated that DLBCL-IF is derived from marginal zone B cells and may represent a large-cell transformation of an underlying MZL [7], however, no direct evidence has been provided to date. The overall survival rate and prognosis of the DLBCL-IF seems to be better than that of a non-IF DLBCL as control group (DLBCL-CG) as the majority of cases present in stage 1 or 2, show significantly lower International Prognostic Index (IPI) scores than the DLBCL-CG [6]. Consequently, it has been previously suggested that DLBCL-IF is a distinct clinicopathologic entity [7].

In the case described here, we provide a direct evidence genetically linking DLBCL-IF and MZL. Furthermore, we identify a novel del(20q12) and a *BIRC3* missense mutation in DLBCL-IF, but not the patient’s preceding MZL involving skin, strongly suggesting that these genomic alterations are at least in part responsible for the large cell transformation.

## Materials and methods

### Histology and immunohistochemistry

Formalin-fixed paraffin-embedded tissue sections were stained with hematoxylin and eosin (H&E). Immunohistochemical stains were performed on 4 μm tissue sections using an Autostainer (Leica BOND platform, Buffalo Grove, IL) according to manufacturer’s instructions. Sections were deparaffinized in xylene and graded alcohols. Detection of the antibodies was performed using a chromogenic substrate, diaminobenzene (DAKO). The following antibodies were used: CD1a, CD3, CD5, CD10, CD20, CD23, CD30, CD79a, BCL6, Oct-2, BOB.1 (all from Leica), CD2, CD8, BCL2, CD45, CD68, CD138, CD163, MUM-1/IRF4, ALK, Ki67, p53 (all from Dako), BCL1 (Fisher), c-MYC (Epitomics), PAX5 and CD15 (both BD Bioscience), CD4 (Biocare), CD56 (Zymed/Invitrogen), CD57 (Thermofisher), PD-1 (Abcam), TIA1 (Immunotech), and panCK (Biogenex),

### Molecular analysis for clonality

DNA was extracted from either fresh tissue (i.e., skin) or paraffin embedded (i.e., lymph node) tissue. PCR amplification was subsequently performed using two sets of fluorescently-labeled primers (InVivoScribe Technologies) that hybridize to a conserved V-framework (i.e., FR2 or FR3) region and the conserved J-region of immunoglobulin heavy chain (*IGH*) gene. The PCR products were size separated by capillary electrophoresis on a 3500xL Genetic Analyzer (Life Technologies). Data were analyzed (GeneMapper v5.0 software) and then reviewed for peak patterns consistent with a clonal expansion.

### Fluorescence in situ hybridization (FISH) analysis

FISH was performed on 3 μm formalin-fixed paraffin embedded sections using the Vysis LSI D20S108 (20q12) SpectrumOrange probe and the Vysis CEP 8 (D8Z2, 8p11.1-8q11.1 alpha satellite) SpectrumGreen (Abbott Molecular Laboratories, Abbott Park, IL), according to the manufacturers’ instructions. In brief, slides were deparaffinized using xylene incubation (×3), followed by ethanol wash steps (100, 70%). Prior to hybridization the slides were treated with Dako Pre-Treatment solution (Dako, Inc K5799) followed by digestion with pepsin (37 °C, 15 min). Slides were dehydrated in ethanol (70, 85, 100%), dried and the FISH probes were added and incubated overnight. The following morning the slides were washed, counterstained with DAPI and manually visualized and scored.

### Gene mutation analysis

Detection of single nucleotide variants (SNVs) and insertions/deletions (indels) analysis of paraffin-embedded skin and lymph node tissue samples was performed by the University of Pennsylvania clinical genomics laboratory, the Center for Personalized Diagnostics. The genes sequenced were part of a custom, targeted next-generation sequencing amplicon panel for 68 hematologic malignancy-associated genes (TruSeq Custom Amplicon, Illumina Inc.) based on previously described analyses [9, 10]. Briefly, individual library preparations were pooled and concurrently sequenced on the Illumina MiSeq (Illumina, Inc.). To assure a minimum read depth of 250×, the mean depth of coverage across the entire panel was 2000×. Detection of SNVs was validated to a 4% allele frequency and large indels to a 1% allele frequency. A separate assay was performed in parallel to sequence the *CEBPA* gene using long range PCR to isolate the gene and library preparation was performed using the tagmentation-based Nextera library preparation kit (Illumina, Inc.). The CEBPA gene was sequenced in tandem with the hematologic next-generation sequencing panel and reported together with the custom heme-NGS panel.

A custom bioinformatics pipeline was utilized to detect alterations [11]. Manual review of the data including visualization of variants was performed on all samples following bioinformatics processing variants, with variants compared with our knowledgebase and on-line databases for further curation, using human reference sequence UCSC build hg 19 (NCBI build 37.1) for comparison. Single nucleotide polymorphisms (SNPs) with a minor allele frequency (MAF) > 0.1% were considered benign and were not reported; these calls were based on the Exome Variant Server (http://evs.gs.washington.edu/EVS), the ExAC browser (http://exac.broadinstitute.org) and dbSNP. Reported variants used nomenclature based on the Human Genome Variation Society nomenclature guidelines (http://www.hgvs.org/mutnomen) and internally categorized into five different categories (benign, likely benign, variant of uncertain significance, likely pathogenic, pathogenic); the categories likely benign, variant of uncertain significance and likely pathogenic were reported as variants of uncertain significance in the electronic health record.

### Flow cytometry

Flow cytometry was performed on representative tissue from the inguinal lymph node using the FACSCantoII flow cytometer from BD Immunosystems. Samples were prepared by aliquoting 50–75 μL of prepared cell suspension into reagent tubes. Twenty microliter of rabbit blocking reagent was added to the B cell clonality tube and allowed to incubate at 37 °C for 20 min and 20 μL of rabbit serum was added to the remainder of the tubes. Laboratory prepared and premixed antibody cocktail was added in 20 μL aliquots to each designated tube and allowed to incubate in the dark for 20 to 30 min. The cells were then washed with 3 to 5 ml of wash buffer that was added to each tube, followed by centrifuging at 1450 rpm for 5 min at 10 °C. The cell pellet was resuspended in 0.5 ml of wash buffer and 10 μL of DAPI working solution was added to each tube. Data is then acquired on the flow cytometer.

## Case presentation

### Clinical findings

A 53-year-old man was evaluated in our Dermatology Clinic due to a 1-year history of generalized itching, fatigue of 2–3 month’s duration, nausea over the past month, and a new left mid back rash. A physical exam was essentially unremarkable except for a 1.2 cm red/purple plaque on the left mid back that was biopsied. Basic blood work was within normal limits. PET/CT was performed and revealed inguinal, pelvic, retroperitoneal, axillary, and cervical lymphadenopathy. The patient was referred to surgery for excisional biopsy of a right inguinal lymph node. Of note, a CT scan performed 4 years earlier had demonstrated inguinal lymphadenopathy (3.8 cm lymph node). No additional workup was done at that time. A follow-up CT scan performed 2 years later showed mildly prominent bilateral axillary lymphadenopathy. One year prior to the current evaluation, the patient noted swelling in the right and left axilla.

### Pathologic findings

#### Skin biopsy

**Fig 1:**
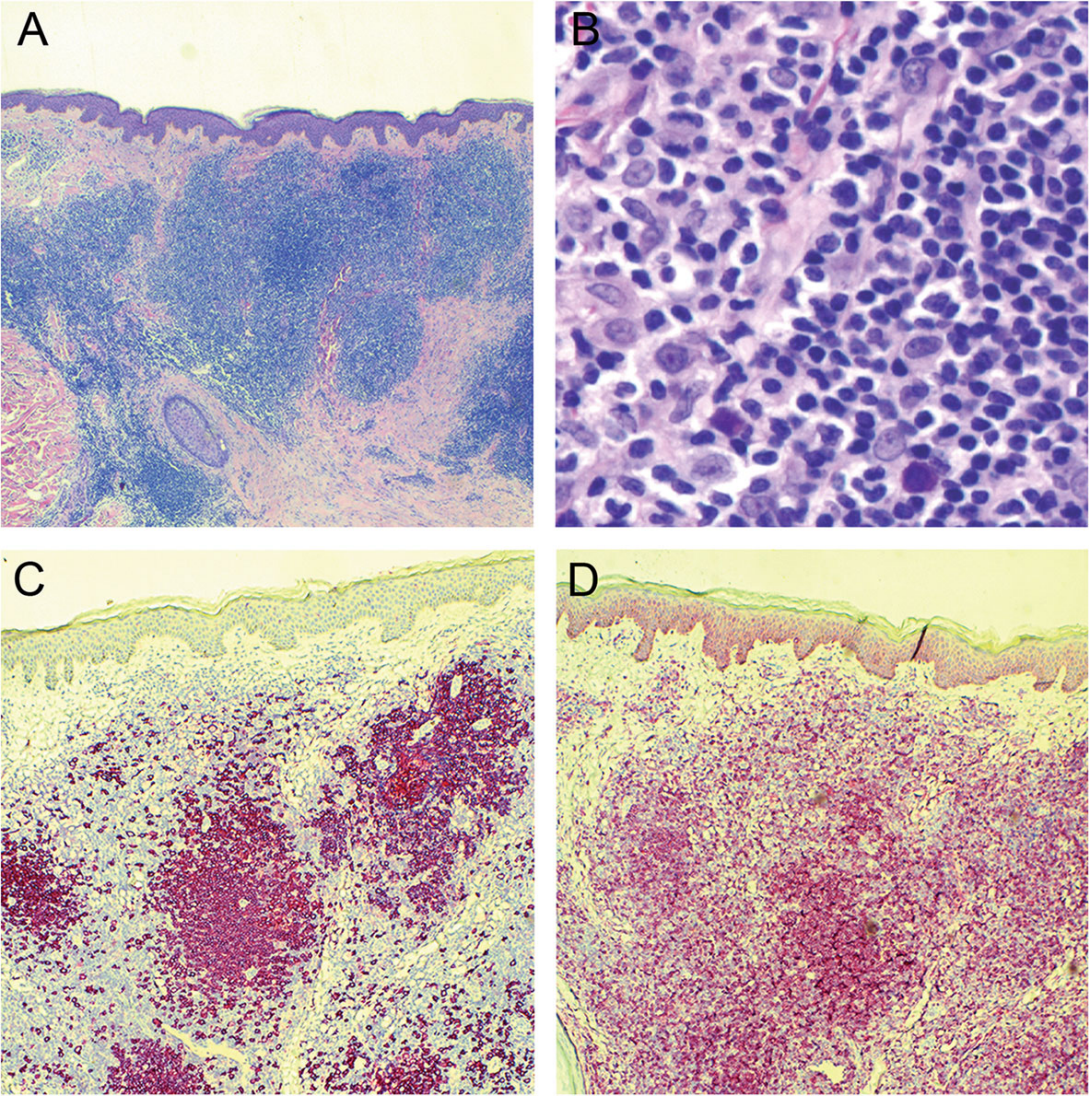
Histological findings of the skin biopsy from initial presentation. **a** Low magnification (25×) demonstrating a nodular infiltrate in the superficial and deeper dermis (H&E). **b** At higher power (400×) the majority of the cells are small and have irregular nuclei and inconspicuous nucleoli. There are scattered large transformed immunoblasts (H&E). **c** CD20 stain highlights the nodules of B lymphocytes (CD20 immunohistochemical stain, 50×) **d** The B lymphocytes in the nodules are positive for BCL2 (BCL2 immunohistochemical stain, 50×)

The H&E-stained sections demonstrated a dense nodular lymphoid infiltrate in the superficial and deeper dermis without involvement of the epidermis (Fig. 1a). The infiltrate was composed of predominantly small lymphocytes with scattered larger transformed cells with immunoblast morphology (Fig. 1b). Immunohistochemical stains demonstrated nodules of CD20 positive B cells around and within follicles, with scattered larger interfollicular B cells also present (Fig. 1c). CD3 stain highlighted abundant interfollicular T lymphocytes (not shown). Essentially all neoplastic B cells as well as many of the interfollicular T cells were positive for BCL2 (Fig. 1d). BCL6 was negative in the tumor cells. The findings were consistent with cutaneous involvement by marginal zone lymphoma.

#### Excisional biopsy of the right inguinal lymph node

**Fig. 2:**
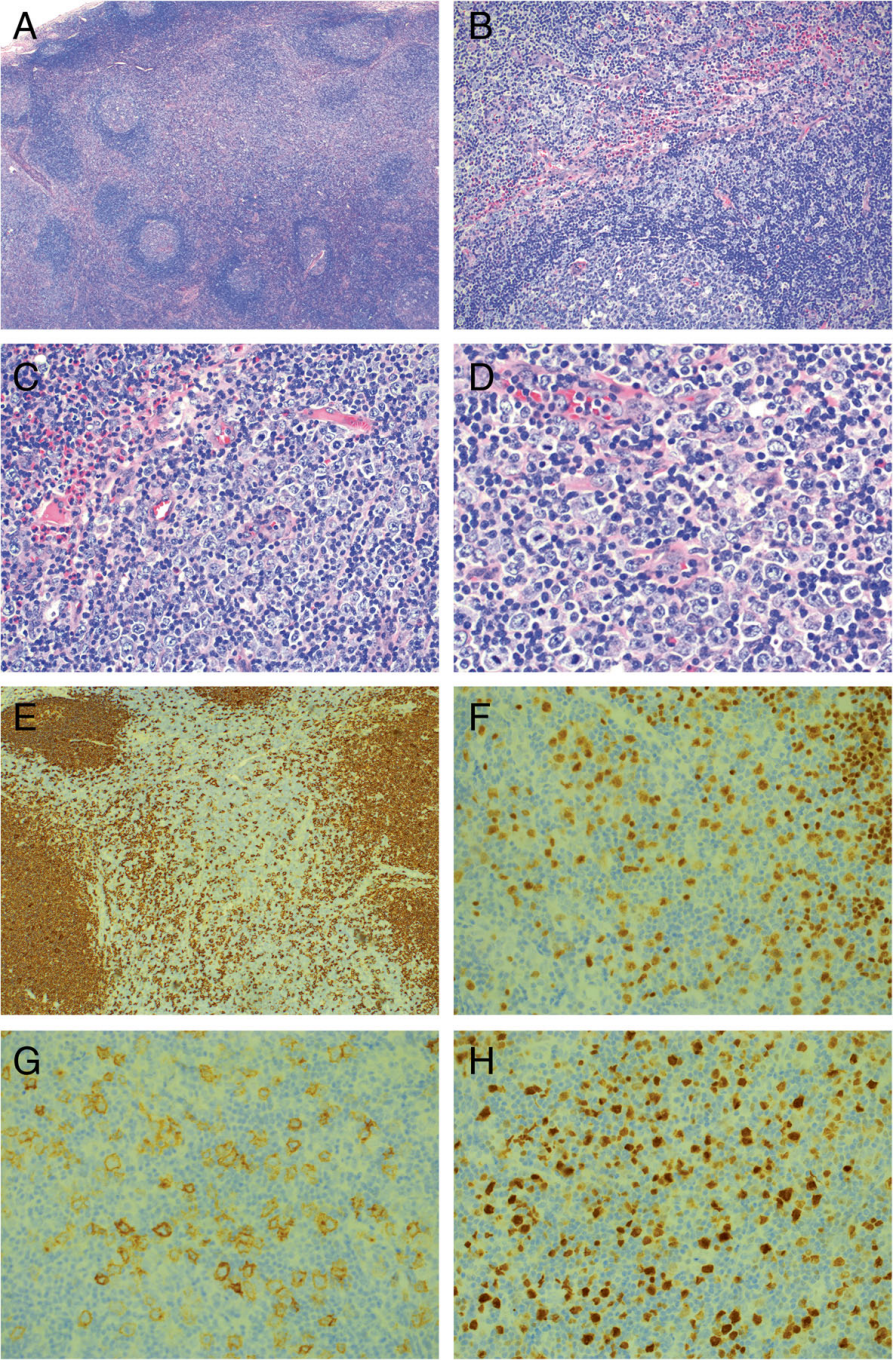
Histological findings of the inguinal lymph node excision 2 weeks after initial presentation. **a** Low-power view (25×) shows a lymph node with a preserved architecture with intact capsule, patent sinuses and polarized germinal centers with well-demarcated mantle zones. The interfollicular areas are expanded (H&E). **b** At higher magnification (100×) the interfollicular infiltrate is composed of numerous large transformed immunoblasts in the background of small lymphocytes, abundant eosinophils, scattered plasma cells and histiocytes (H&E). **c** At 200× magnification (H&E) and (D) at 400× magnification the large atypical cells have large oval to slightly irregular nuclei with vesicular chromatin, prominent nucleoli and scant to moderate amounts of clear to amphophilic cytoplasm (H&E). **e** The large atypical cells are immunoreactive for CD20 (CD20 immunostain, 50×) as well as for **f** PAX5 (PAX5 immunostain, 200×), **g** CD30 (CD30 immunostain, 200×) and **h** MUM1 (MUM1 immunostain, 200×)

The H&E stained sections showed a lymph node with an overall well-preserved nodal architecture including reactive lymphoid follicles with well-demarcated mantle zones (Fig. 2a). The interfollicular zones were, however, expanded by numerous large atypical mononuclear cells admixed with abundant eosinophils, small lymphocytes, plasma cells and histiocytes (Fig. 2a and b). The larger cells had oval to slightly irregular nuclear contours, vesicular chromatin, prominent nucleoli, and scant to moderate amount of clear to amphophilic cytoplasm (Fig. 2c and d). Immunohistochemical stains showed that the large cells were positive for B-cell markers CD20 (Fig. 2e), PAX5 (Fig. 2f), and CD79a, as well as BCL6 (subset), CD30 (Fig. 2g), MUM1(Fig. 2h), CD23, c-MYC (40–50%), p53 (dim in 40% of the large cells), BOB.1 (variable) and Oct-2 (variable). The Ki-67 proliferation index in the interfollicular areas was high, exceeding 70%. Key negative stains included BCL2, CD10, and CD15. CD138 highlighted plasma cells with a kappa to lambda ratio of 3:1. An in-situ hybridization study for Epstein-Barr virus (EBER) was negative. CD20, PAX5 and CD79a stains also marked the abundant reactive follicles with CD10+, BCL6+ and BCL2- germinal centers and BCL2+ mantle zones. The proliferation index within the germinal centers was high as expected (>90%) and highlighted the polarized light and dark zones. The infiltrating small T lymphocytes were positive for CD2, CD3, CD5 and CD7 (major subset). The CD4 to CD8 ratio was increased (>5:1). CD57 and PD-1 were positive in a subset of germinal center T cells whereas TIA1, perforin, and granzyme B were positive in scattered small interfollicular T cells with a pattern similar to CD8 staining. CD56 and ALK-1 stains were negative. CD68, CD163, and CD1a stained scattered histiocytes and accessory cells respectively.

#### Ancillary studies on the lymph node specimen

**Fig. 3:**
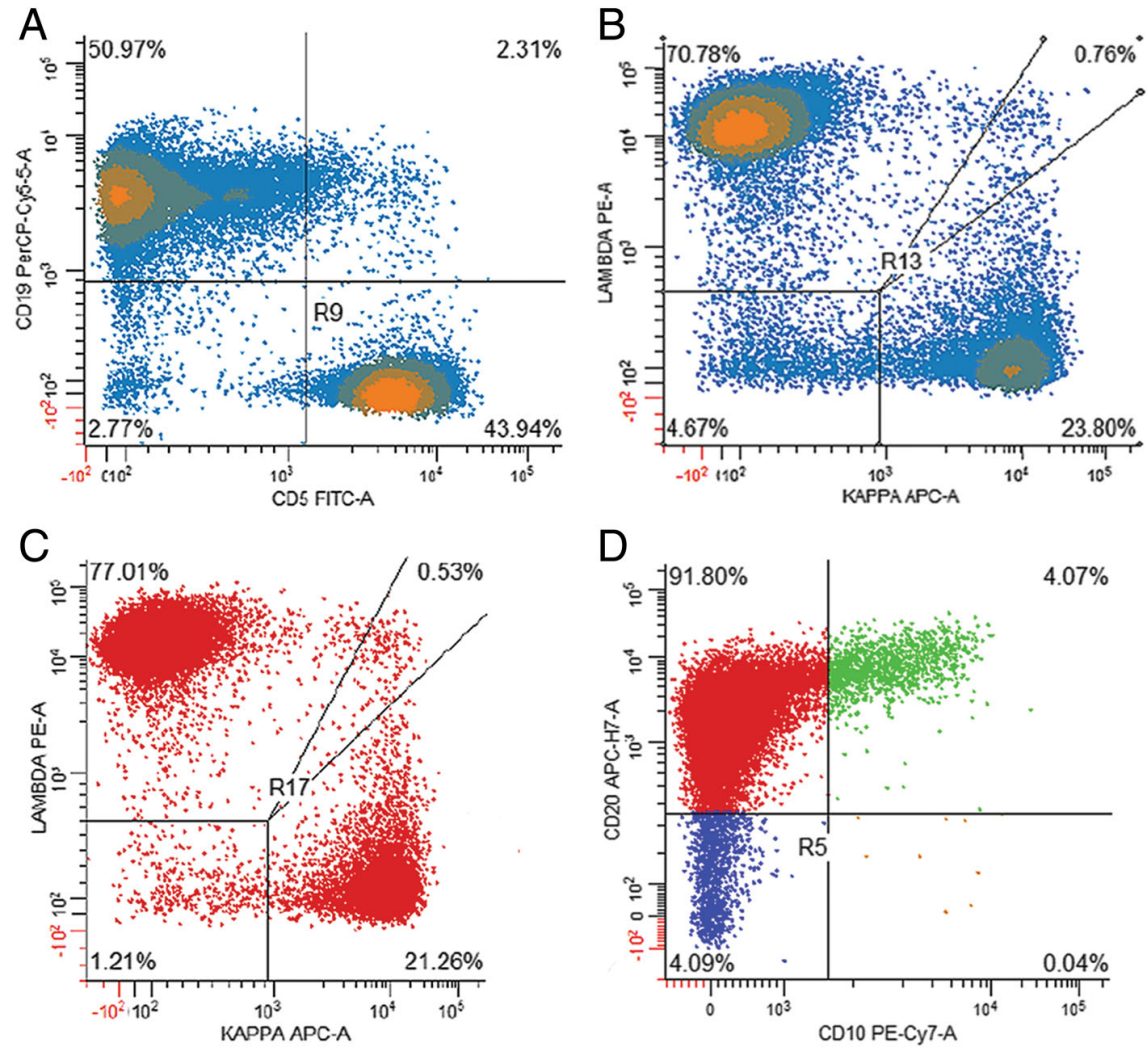
Flow cytometric analysis of representative tissue from the inguinal lymph node. **a** The CD19/CD5 plot demonstrates and admixture of CD19-positive B cells and CD5-positive T cells. **b** Gating on the CD19 positive B cells demonstrates a clear lambda bias. **c** the lambda bias of is also seen when gating on CD20 positive B cells. **d** The majority of the CD19 positive B cells were found to be CD20 positive and CD10 negative. The presence of a subset of CD10 positive B cells and subset positivity for kappa light chain staining is compatible with germinal center B cells that are admixed with the malignant B cells

Flow cytometry studies were performed on representative tissue from the right inguinal lymph node. As shown in Fig. 3a–c, there was a large population of CD19+ B lymphocytes (51% of all lymphoid cells) with a strong lambda bias bordering on overt restriction (kappa: lambda ratio of 0.33 and 0.27 for the CD19+ and CD20+ cells, respectively). Minor subsets of all B lymphocytes were CD5+ (Fig. 3a) or CD10+ (Fig. 3d); these cells expressed predominantly kappa and, hence, were not part of the apparent lambda clone.

**Fig. 4:**
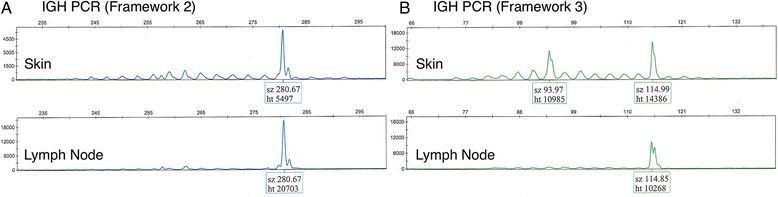
*IGH* PCR analysis of both specimens revealed a shared pattern of peaks. **a** Primers for FR2 region identified a clonal peak at approximately 281 bp in the skin and the lymph node. **b** Similarly, a reproducible peak at approximately 115 bp was identified with primers targeting the FR3 region of *IGH*. An additional 94 bp peak was identified in a polyclonal background in the skin but not lymph node specimen

Molecular studies for *IGH* gene rearrangement performed on the skin biopsy showed a 281 base pair (bp) peak in framework 2, as well as 94 bp and 115 bp peaks in framework 3 (Fig. 4). Examination of tissue obtained from the right inguinal lymph node demonstrated the same 281 bp peak in framework 2 and the same 115 bp peak in framework 3 whereas the 94 bp peak seen in the skin specimen was not seen in the lymph node (Fig. 4b). The overall results indicate that the lymphoproliferative disorders involving lymph node and skin are clonally related. The significance of the 94 bp peak is unclear, but it may represent a separate (sub-)clonal expansion. No clonal T-cell receptor (TCR) rearrangement was detected in the lymph node specimen.

**Fig. 5:**
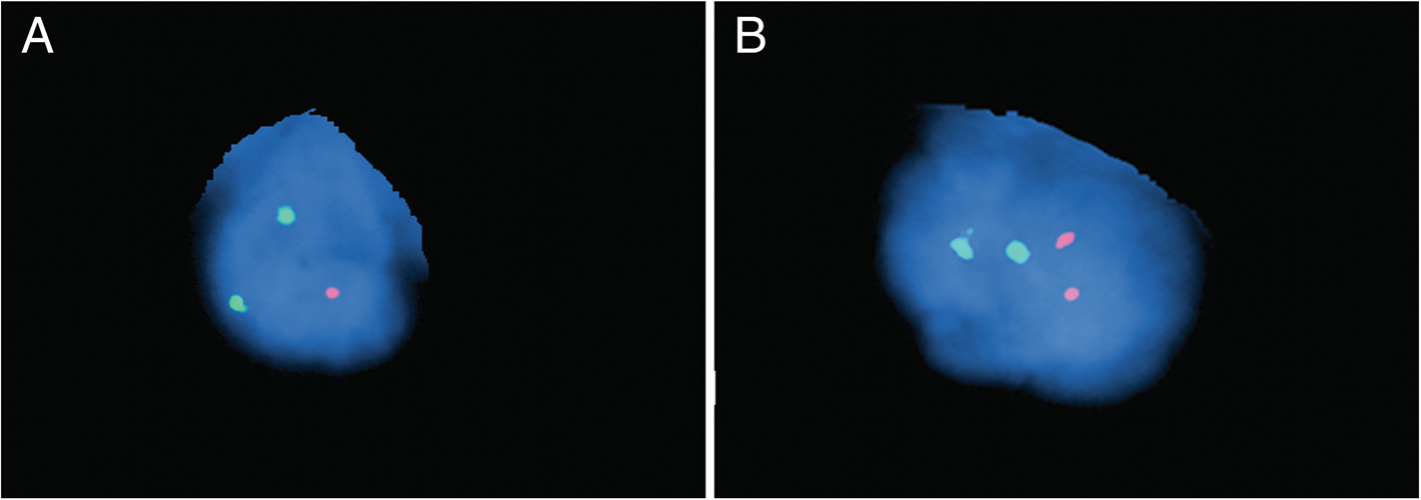
FISH analysis of the lymph node specimen. Representative images of FISH analysis of the lymph node specimen using probes specific for chromosome 20 (20q12, red) and centromere of chromosome 8 (green). **a** Cell positive for del(20q) and **b** cell negative for del(20q)

FISH studies for rearrangements of MYC, BCL2, and BCL6 performed on the lymph node were also negative. Previous report described del(20q) in MZL with aggressive features [12]. Since this patient’s DLBCL was thought be derived from a MZL, additional FISH analysis for a 20q12 deletion was performed on both skin and lymph node material to determine whether this abnormality may be present. The analysis revealed that 38/150 (25.3%) of cells in the involved lymph node were harboring del(20q12) (Fig. 5), whereas the skin lesion was negative for this deletion (data not shown).

Targeted DNA sequencing studies using our hematologic malignancy panel on the lymph node tissue did not reveal any known disease-associated mutations, however it did identify two variants of uncertain significance (Table 1): a missense variant in *BIRC3* (Baculoviral IAP repeat-containing protein 3) at amino acid 572 converting the wild type residue, cysteine, to tyrosine in *BIRC3* (p.C572Y → c.1715G > A) in 491 reads out of a total 9155 sequence reads for an allele frequency of 5.36% and a missense variant in *EZH2* gene (Enhancer of Zeste Homolog 2) at amino acid 322 converting the wild type residue, Asparagine, to Serine (p.N322S → c.965A > G) in 818 reads out of a total 1561 sequence reads for an allele frequency of 52.40% . Subsequent sequencing studies of the skin revealed the same missense variant in *EZH2* as in the lymph node in 1097 reads out of a total 2165 sequence reads for an allele frequency of 50.67% but no other alterations, including the *BIRC3* mutation, were identified.

**Table 1:** Summary of identified missense variants in the lymph node and skin biopsy specimens

Gene alteration	Protein change	Nucleotide change	Frequency in lymph node	Frequency in skin
*BIRC3*	C572Y	c.1715G > A	5.36%	0%
*EZH2*	N322S	c.965A > G	52.40%	50.67%

*Abbreviations: A* adenine, *C* cysteine, *c.* coding, *G* guanine, *N* asparagine, *p.* protein, *S* serine, *Y* tyrosine

## Discussion

We report a rare case of a DLBCL with an interfollicular pattern of involvement (DLBCL-IF) with sparing of the nodal architecture including lymphoid follicles. This pattern is common in peripheral T-cell lymphoma, not otherwise specified (PTCL, NOS) that shows proliferation of malignant cells in the paracortex [1]. Our case showed that the large malignant cells were admixed with an inflammatory infiltrate including abundant eosinophils. It has been reported that approximately 60% of DLBCL-IF cases contain reactive inflammatory cells that are admixed with the large cells, a feature that is frequently seen also in PTCL, NOS [1, 6, 7]. Thus, DLBCL-IF can mimic histological picture of PTCL, NOS and it is of utmost importance to be aware of this entity, as PTCL-NOS carries a worse prognosis than DLBCL-IF and calls for different therapeutic approaches. Therefore, immunohistochemistry studies are required to differentiate between these two entities. It is important to keep in mind that both these types of lymphoma can be immunoreactive for CD30. Furthermore, it is critical to always consider DLBCL-IF in the differential diagnosis of atypical interfollicular proliferations. Since distinguishing it from a reactive interfollicular hyperplasia may be sometimes difficult, given the abundant background of inflammatory cells often seen in this type of lymphoma, additional ancillary testing including *IGH* gene rearrangement studies and flow cytometry may prove particularly useful to establish the diagnosis.

It has been postulated that DLBCL-IF may be derived from marginal zone B cells and may represent a large-cell transformation of a MZL [7, 8]. To date, however, there was no direct evidence to confirm this hypothesis. Here we show for the first time a direct link between a MZL and DLBCL-IF by demonstrating the presence of the same clonal *IGH* rearrangement in the patient’s skin and nodal biopsies. Furthermore we identified del(20q12) in the DLBCL-IF. This genetic abnormality has not been reported to date in a DLBCL of any kind. Deletion of chromosome 20q is a common cytogenetic abnormality in various myeloid neoplasms, most notably myelodysplastic syndrome [1, 13, 14] but its association with lymphoid malignancies is rare and limited to a few reports. This includes a case of lymphoplasmacytic lymphoma/Waldenström macroglobulinemia [15] and two cases of marginal zone lymphoma [12, 16]. Another study of 64 patients with chronic lymphocytic leukemia (CLL) revealed that del(20q) appears to be a therapy-related abnormality in CLL involving both myeloid and lymphoid cells and may represent disease progression [17, 18]. Given that del(20q12) in this patient was identified in the lymph node and not skin lesion and that the patient has not received any prior chemotherapy at that time, this abnormality is likely related to transformation of MZL to DLBCL and implicates a loss of important gene(s) likely with tumor suppressor function. Potential candidate genes that map to this region include *MYBL2* [19], *Dido* [20], *L3MBTL1* [21] and *SAMHD1* [22].

Next-generation sequencing studies using a custom panel of 68 hematologic malignancy-associated genes revealed the same missense variant in *EZH2* in both the skin and lymph node specimen and a missense variant in *BIRC3* that was present only in the lymph node specimen. The *EZH2* variant was listed in all three databases searched at very low allele frequencies (5 cases in COSMIC, in 4/13002 alleles in the exome variant server, and 37/121,370 alleles in the ExAC database).

The missense variant in *BIRC3* (p.C572Y → c.1715G > A), not reported in any of the three databases searched (see methods section), was detected only in the lymph node but not skin specimen at an allele frequency of 5.36%. Morphologic examination revealed that the tumor comprises about 10-15% of the lymph node specimen. When these two observations are taken together, the data imply that the *BIRC3* mutation may be present in a heterozygous state within the neoplasm (i.e., a tumor burden of approximately 10% with a heterozygous mutation would be expected to generate an allelic burden of approximately 5%). So, even though this variant has not been previously described, the circumstantial evidence implies this is an oncogenic mutation that occurred in the malignant DLBCL-IF cells.

The fact that this mutation was only seen in the lymph node and not in the skin biopsy strongly suggests that it may have contributed to transformation of this patient’s MZL to DLBCL. *BIRC* mutations are not among the recurrent mutations in the “standard” DLBCL according to a recent report [23]. *BIRC3* gene located on chromosome 11 at 11q22.2 encodes cIAP2 (cellular inhibitor of apoptosis 2), that acts as an E3 ubiquitin ligase that regulates NF-kappa-B signaling and affects a variety of cell functions including apoptosis, proliferation and immune modulation [24, 25]. Of note, proteins from the IAP family are potential therapeutic targets in hematological malignancies [26]. *BIRC3* mutations have been identified in various small B cell lymphomas including chronic lymphocytic leukemia (CLL) and splenic marginal zone lymphoma (SMZL) [27, 28]. More recent studies demonstrate the role of the ubiquitin ligases, both cIAP2 and a closely structurally related cIAP1, encoded by the *BIRC2* gene, in NF-κB signaling triggered by the B cell receptor (BCR) in DLBCL cells [29].

The alteration in *EZH2* gene (p.N322S → c.965A > G) was observed with an allele frequency of approximately 50% in both specimens and this most likely represents a benign/low-oncogeneic potential polymorphism rather than a highly oncogenic mutation. *EZH2* gene on 7q36 encodes the histone methyltransferase which initiates epigenetic silencing of genes [30] and plays a role in normal B cell development [31]. *EZH2* is mutated in a variety of tumors including lymphomas and targeting of EZH2 protein with specific inhibitors show promising results in preclinical studies and early clinical trials [32–34]. Interestingly, it has been reported that *EZH2* polymorphisms may contribute to susceptibility of tumor development and predict overall survival in some cancers [35–38] and may have also contributed to progression of the disease in this patient.

## Conclusions

In summary, we present a rare case of diagnostically challenging DLBCL with an interfollicular pattern and provide data that support the hypothesis that DLBCL-IF can arise from a MZL. To our knowledge, this represents the first reported case where DLBCL-IF and MZL are shown to be clonally related. We identify a novel del20q and a *BIRC3* missense mutation in this patient’s DLBCL-IF that have not been previously reported in DLBCL. Given the absence of both these genetic abnormalities in the patient’s MZL, they both may have contributed to the large cell transformation.

## Abbreviations

ABC DLBCL: Activated B-cell-like DLBCL
BCR: B-cell receptor
BIRC3: Baculoviral IAP repeat-containing protein 3
cIAP2: Cellular inhibitor of apoptosis 2
CLL: Chronic lymphocytic leukemia
CT: Computed tomography
del(20q): Deletion 20q
DLBCL: Diffuse large B-cell lymphoma
DLBCL-IF: Interfollicular diffuse large B-cell lymphoma
EZH2: Enhancer of zeste homolog 2
FISH: Fluorescence in situ hybridization
GCB DLBCL: Germinal center (GC) B-cell-like DLBCL
IGH: Immunoglobulin heavy chain
IPI: International prognostic index
MZL: Marginal zone lymphoma
PET: Positron emission tomography
PTCL, NOS: Peripheral T-cell lymphoma, not otherwise specified
WHO: The World Health Organization
